# Perceptions of abused Chinese women on community-based participatory approach programme in addressing their needs

**DOI:** 10.1080/17482631.2024.2331107

**Published:** 2024-04-02

**Authors:** Elaine Hoi Yee Chow, Agnes Tiwari

**Affiliations:** a School of Nursing, Tung Wah College, Hong Kong, China; b School of Nursing, Hong Kong Sanatorium & Hospital Limited, Hong Kong, China

**Keywords:** Intimate partner violence, community-based participatory research, perceptions, experiences, qualitative, abused women

## Abstract

**Background:**

The community-based participatory approach (CBPA) has gained increasing recognition worldwide for enhancing the effectiveness of intervention. It is relatively new in Chinese societies and participants’ perceptions are underexplored. This study aims to explore abused Chinese women’s perceptions on the CBPA programme in addressing their needs.

**Methods:**

A total of 11 abused Chinese women were recruited for a focus group and individual interviews. A semi-structured interview guide was used. All interviews were audio-recorded and data were transcribed verbatim. Conventional content analysis was used for analysis.

**Results:**

Four themes were identified regarding the women’s perceptions and experiences of the community-based participatory approach programme: (1) Women’s perceived acceptability of the CBPA programme; (2) Women’s perceived usefulness of the CBPA programme; (3) Women’s perceived feasibility of the CBPA programme; and (4) Empowering the women through participating in CBPA.

**Conclusions:**

Abused Chinese women had high perceived acceptance and positive experiences towards the community-based participatory approach. Women benefited from their robust participation throughout the process. The findings confirm the potential of using the community-based participatory approach in designing interventions for future programme planning and intervention to address the needs of abused Chinese women.

## Introduction

Intimate partner violence (IPV) is a significant public health problem across cultures, ethnicities and religions (Ellsberg et al., 2008; Garcia-Moreno et al., 2006). A multi-country study conducted by the World Health Organization indicated that the lifetime prevalence of intimate partner violence experienced by women varied from 15–71% and includes physical, sexual and psychological violence (Garcia-Moreno et al., 2006). Despite various forms of interventions (Hameed et al., 2020; Rivas et al., 2019) existing for abused women, a high prevalence (Garcia-Moreno et al., 2006) and destructive consequences continue to affect women.

Current interventions for abused women are a top-down approach, relying heavily on the decisions of researchers who are often outsiders; abused women have little involvement in the process. Due to the uniqueness of each abused woman, standardized intervention could not fully address the concerns of abused women and might lead to an ineffective result (Zomahoun et al., 2019). A more effective and tailored intervention could bring benefit by adopting a community-based participatory approach (CBPA) in the intervention development process. However, there is a paucity of evidence indicating a CBPA is appropriate to meet the needs of abused Chinese women and their experiences throughout the process.

Given the high prevalence rate and significant impacts of IPV (Ellsberg et al., 2008). A bottom-up, tailored intervention is essential for addressing the women’s concerns and this could be achieved by robust engagement of the abused women during the process of intervention planning and implementation (Cyril et al., 2015; Teresa et al., 2023). CBPA is an approach to research that can strengthen the design and enhance the appropriateness of intervention. Moreover, CBPA highlights power sharingand equitable partnerships that could empower the community stakeholders, increase the sustainability and ultimately improve the outcomes of the programme (Israel et al., 2010). CBPA has been well documented for increasing the effectiveness of the health related programme to improve participants’ overall health and well-being. Numerous studies have demonstrated the success of incorporating CBPA in interventions in a variety of contexts and across different populations (Foster et al., 2010; Lin et al., 2019; Ma et al., 2012). CBPA has been well known as a research paradigm recommended for vulnerable populations to reduce health disparities and aid in developing culturally specific interventions for minority groups in the past decades (Suarez‐Balcazar et al., 2020; Vaughn et al., 2017). Abused women is one of the health disparities groups which deserve more attention as they play an important role in families.

For instance, CBPA was adopted as an intervention for reducing the prevalence of intimate partner violence (IPV), to identify the women’s needs and developa culturally appropriate instrument to measure women’s IPV experiences in Western countries (Bloom et al., 2009; Bright et al., 2018; Burke et al., 2013; White et al., 2013). Current studies have focused on evaluating the effect of intervention quantitatively (Kobeissi et al., 2011; Wieland et al., 2013) and explored the perceptions of academics or community services providers regarding the challenges in CBPA (Katigbak et al., 2016; Pivik & Goelman, 2011). The perceptions and experiences of abused women on a CBPA programme remains unexplored.

Developing a culturally sensitive intervention for abused women demands urgent attention. Adopting CBPA to guide intervention development for abused Chinese women was extremely limited. To my knowledge, a CBPA programme tailored to abused Chinese women has been implemented in Asian countries to address their multi-faceted needs. Despite abundant evidence showing CBPA is a useful and effective methodology, little is known about the women’s perceptions of adopting CBPA in IPV intervention and there is an urgent need to have an in-depth understanding of whether CBPA is practically sensible and able to provide significant benefits for the abused Chinese women to address this complex public health issue. Therefore, this study employs a qualitative approach to explore the perceptions of abused Chinese women on a CBPA programme as well as their experiences in CBPA.

This study was the first study to discover the abused Chinese women’s experiences and perceptions throughout their participation in the CBPA programme. Abused Chinese women’s perception on CBPA in addressing their needs will be explored for future development of a woman-centred and tailored intervention that addresses their needs with culture elements.

## Method

### Aim

The aim of this study was to explore the abused Chinese women’s perception of the CBPA programme and their experience of participating in this approach.

### Design

A qualitative descriptive approach was adopted in this study in order to gain an in-depth understanding of the perceptions of abused Chinese women who had participated in a CBPA intervention programme (Table I) and their experiences throughout the process (Creswell & Poth, 2016). Qualitative study is a research methodology that allows exploration of deeper insights of an individual, thus helping to gather women’s experiences and perceptions and answer the why and how (Denzin & Lincoln, 2011).

**Table I.** Brief description of the CBPA programme for abused Chinese women.

	Brief description of the component	Venue	Duration	Time
Relaxing Singing	One of the favorite stress releasing activities of the women. It brings them relaxation and happiness. Opens up a bridge for the women to get to know each other and break the ice for future activities in the programme.	Indoor Karaoke center in Tsuen Wan	4 hours	11:00–1500
Brain Gym Group	A kind of mindfulness exercise which involves some simple movements to integrate the whole brain, senses and body. This exercise allows the women to minimize painful and frightening feelings; giving women a sense of mastery over their thoughts and feelings.	Community Centre in Kwai Chung	3 hours	10:00–13:00
Relaxing Daytrip	A kind of parent–child activity, it is an outdoor day trip in Lamma Island to nurture intimacy in the family.	Lamma Island	One day	0830–1700
Cleansing Enzyme Workshop	This is a kind of diversional therapy class and personal development activity. Some women were the tutor’s assistant, actively participating from preparing teaching materials to delivering the class.	Community Centre in Kwai Chung	3 hours	10:00–13:00

The CBPA intervention programme consisted of four components, the contents of which are listed in Table 1.

### Participants

The study was conducted in a community centre which provided diversified social and health related services for people in different age groups in Hong Kong. Purposive sampling was used, women were eligibe if they were Chinese, aged over 18, had an intimate relationship in the preceding 12 months, lived in Kwai Chung district, were screened as abused by intimate partner according to the five-item Chinese version of Abuse Assessment Screen (AAS) (Tiwari et al., 2007) and either had joined the CBPA programme or participated in developing the CBPA programme. Women who were unable to communicate in Cantonese or Putonghua, had a history of psychotic illness or were unable to give informed consent were excluded from the study.

### Data collection

Women’s perceptions of the CBPA intervention programme and their experiences were elicited through a face-to-face interview with the helped of an interview guide. There were open-ended questions and follow-up questions, and probes were included to elicit more in-depth and relevant opinions. The interview guide was reviewed by an expert in the field of intimate partner violence in order to ensure the content validity.

During the interview, a social worker at the community centre who had established a good relationship with the participants stayed in the room throughout the whole interview, allowing the women to express freely. Extra effort was made to ensure a comfortable interview, which was started by using narratives to let the women express their thoughts and opinions. “How’s the CPBA programme?”, “Would you invite your friends to join if it was held again in the future?” These questions helped to explore the women’s thought regarding the CBPA programme, then the women were probed by asking, “Could you tell me more about this?” Women were invited to elaborate why they liked and which component they liked the most or impressed them most. To further explore the perceptions of the programme, women were prompted by asking, “Are you benefitting from it? if any, how has the programme been a benefit to you? on which aspect?” They were encouraged to elaborate more by asking, “Tell me which component helps you most and how does it help you? Any example?” These questions helped to better understand how the women perceived the CBPA programme. To further explore women’s experiences throughout the process of CBPA from planning to implementation, they were asked, “As you have taken part in the process of the programme development, would you please share your experiences and feelings during your participation?”

To ensure privacy, all interviews were conducted in a private room. All interviews were conducted by the principal investigator who was a female nurse and a nurse educator. All interviews were audio-taped and field notes were kept to record the facial expressions and emotions of the participants.

### Ethical considerations

Ethical approval was obtained from the Institutional Review Board of a university in Hong Kong. A notice was posted in the community centre to attract potential participants. All eligible participants had the purpose of the study, potential risks and benefits explained to them individually. Potential participants were reassured that they could withdraw from the study at any time with no adverse effects and without giving a reason and a signed written consent form was obtained.

### Data analysis

Data analysis was conducted after each interview to ensure the content of the interviews was understood and deeper understanding could be obtained in a subsequent interview. All interviews were audio-taped and listened to by researchers repeatedly in order to familiarize themselves with the content. Audio-tapes were transcribed verbatim and were read repeatedly, which allowed the researchers to obtain a sense of the whole and comprehend the data (Speziale et al., 2011). Conventional content analysis was used in the study because there were no preconceived categories imposed before data analysis (Hsieh & Shannon, 2005). Key words and phrases were identified and highlighted and notes were made while reading the text to capture the key concepts and derive appropriate codes. Codes were labelled and then sorted into different sub-categories according to their concepts. Sub-categories were then combined and organized into categories. Similar categories were gathered to form themes (Graneheim & Lundman, 2004; Hsieh & Shannon, 2005). Researchers discussed the emergent themes, categories, sub-categories and codes until consensus was reached and rich data was derived from the contexts unil data saturation. Content analysis was used in data analysis which helped to provide an understanding of using CBPA in abused Chinese women.

### Rigour

To ensure the trustworthiness of the study, two researchers read the transcribed text and coded it independently for comparison and then discussed the emergent themes, categories, sub-categories and codes until a consensus was reached in each case. The two researchers were involved in data collection and data analysis, had continuous reflection throughout the process, adopted reflexivity, and reflected on their experience as a nurse and expert researcher in the field of intimate partner violence. Inquiries into abused women’s experiences allows the production of knowledge through reflexive peer discussions (Bradbury‐Jones, 2007; Speziale et al., 2011). Reflexive diaries were kept as a record of researchers documented observations, feelings, thoughts, reflections on assumptions and biases to assure credibility. In addition, prolonged engagement was employed, where researchers immersed themselves in the context of the study, spent sufficient time to observe various aspects, built trust and rapport with the participants, and participated in the whole CBPA programme with the women (Kyngäs et al., 2020).

## Findings

Eleven women were interviewed;two face-to-face semi-structured focus group interviews and one individual interview were conducted. Women ranged in age from 33 to 64 years (Table II) and each session lasted for around 60 minutes. Content analysis revealed four themes to illustrate Chinese women’s perceptions and experiences of the CBPA programme. The four themes are (1) Women’s perceived acceptability of the CBPA programme; (2) Women’s perceived usefulness of the CBPA programme; (3) Women’s perceived feasibility of the CBPA programme; and (4) Empowering the women through participating in CBPA.

**Table II.** Demographics of the participants.

	*N*=11
Age categories	
18–25	0
26–36	2
36–45	4
> = 46	5
Number of children	
1	1
2–3	10
> = 4	0
Being employed or not	
Yes	5
No	6
Marital status	
Married	10
Divorced	1
Country of origin	
Mainland China	5
Hong Kong	6

### Theme 1: Women’s perceived acceptability of the CBPA programme

The CBPA programme was highly praised by the women. Women were satisfied with each component of the programme, they were excited when sharing and recalling every detail and moment. Women asked for the next schedule of this programme, expressed their enjoyment in the programme and gave a lot of suggestions for the next round. One woman asked repeatedly on the next schedule:

When will this programme hold again? It is very good and vivid, I really enjoyed it, I am so glad that I have the chance to join it, remember to call me next time. (N05)

Moreover, women suggested to add more components to increase the diversity of the programme, particularly family-related activities. They also suggested to lengthen the duration of the programme. Women mentioned that they loved every single component in the programme, as explained by two women:

I prefer to have five instead of four components. The family outing was very nice and I hope that more family activities or outings could be arranged. (N04)

The content of the programme was quite suitable for me, there are several components in the programme, those activities were exactly what I want, it was a very good experience for me … (N09)

Women appreciated the idea of adopting a community-based participatory approach, they treasured the opportunity to voice their opinions and took part in planning, organizing and implementing a CBPA programme. The women not only praised the idea of CBPA and the content of the CBPA programme, they valued the partnership concept in CBPA.

I think this was a new attempt. I had never heard this before; this idea was good. If we are allowed to give suggestions and participate in the whole process, I am sure it will be very great because our proposals are what we want and what we need. (N02)

### Theme 2: Women’s perceived usefulness of the CBPA programme

Women gained numerous perceived benefits from the programme such as individual’s physical and psychological health status, harmonious relationships in the family and social networks with women who shared a similar background.

#### Benefits to women’s individual’s health

Many of them expressed that they had insomnia and were under high levels of stress, they felt relaxed after joining the CBPA programme, both physical and psychological well-being were improved, as described by the women:

I have insomnia, I cannot sleep without medication because my relationship with my husband is not good and my children are not good as well. I am under high level of stress. This programme did help me, I could have a break and forgot the unhappiness for a while… (N10)

I feel happier after joining the programme. I feel less stressed and have better relationship with my family. I had less conflict and less arguing with my husband, even when he scolds me, I just kept my mouth off and not to response to him… (N01)

Women mentioned that their emotions were influenced by peers, they learnt from peers who had an optimistic mind. Positive emotion was transmitted through peers, whose positive way of thinking allowed them to achieve a better psychological well-being.

When I see other women looking so cheerful and thinking so positively, I said to myself ‘Why can’t I be like them?’… I got inspired by their positive energy… (N03)

I was impressed by a woman in the programme, she is my friend now … she looks so energetic and she always smile, however, I just knew her situation that was much poorer than mine, so I think I should be more optimistic … (N08)

#### Benefits for family relationships

Resilience of the women was enhanced and they were more able to confront challenges and handle unpleasant issues optimistically after joining the programme. Stable emotion is essential to maintain family harmony and the women explained that it was vital to relieve their stress in order to stabilize their emotions. It was crucial to maintain a good emotional state as a better mental attitudewould benefit the whole family.

I think our (woman and her husband) emotions affect each other. For example, when he comes home and notices that I am in a bad mood or a bad temper, then he will behave and sound terribly bad… however, if I have better mood then less conflict in the end, so it is important to keep my emotions in check… (N07)

The CBPA programme allowed the women to think more positively, it helped them to overcome challenges and aided family relationships and it was cyclical. Improving the individual’s emotional status led to them being more adaptable in handling any conflict with their husband as they were calm enough to face every challenge; as a result, this enhanced husband–wife relationships, as described by the women:

I think the programme did make me to become a more positive person. My stress was relieved and I was in a better emotional state, after joining the programme, I became more cheerful and nonjudgmental, I have less arguing with my husband because I can handle the conflict in a more flexible way. (N11)

I have less conflict with my husband recently. I made new friends from the programme… I could chat and share my thoughts and feelings, it was great. I could keep this mood even back home… my husband asked me why I was smiling all the time. *(N08)*

Women were impressed by the family component of the CBPA programme (an outdoor day trip), they described that they had unforgettable experiences and valuable opportunities to spend time with their family. A special atmosphere was created, which encouraged more quality interactions among family members which was lacking in their family.

I have a lot of good memories from the day trip – I like to go on outings with my husband and son, as we seldom do this. Also, I liked going out with other families, we talk and laugh together, and I am sure my husband quite enjoyed the day trip and we all had a memorable time with our family without arguing and swearing… (N05)

In the past, I forced my husband to join family gatherings, but he agreed to go with me without any hesitation this time. My husband, my son and I enjoyed the day trip so much, I would like to say thank you, and it really creates a magic moment for our family. (N08)

#### Benefits to social networks

Women mentioned the importance of friends as they could share feelings and gain mutual support. Social activities allowed them to share their thoughts, exchange information and support each other. The women emphasized the importance of having friends, especially on weekdays when they particularly felt upset easily because their husband goes to work, son or daughter goes to school, leaving them alone in the house.

It is very important to have friends because feeling cared about by someone else can make me feel comfortable, we (the women) are supporting each other… otherwise I think I would have gone mad if there is nobody to chat with me, especially when my husband has gone to work or sometimes, he swore at me… I felt sad and I need someone to talk to… (N05)

Women who were mainland Chinese immigrants expressed that they had no relatives in Hong Kong, they could only rely on support from peers. The company of peers was crucial, the programme provided a platform and created opportunities for them to build up friendships and exchange community resources information and expand their social networks, as recounted by them

As I came from mainland China, I have no relative in Hong Kong, having friends is important to me because I can share my happiness with them, I can talk to them if I feel unhappy and even share something from the bottom of my heart. Apart from sharing the up and down, we will message each other if we receive any new information from the school or the community centre… It was good that we could meet regularly in this programme. (N11)

### Theme 3: Women’s perceived feasibility of the CBPA programme

There are some factors which encouraged or discouraged the women to join the CBPA programme and their adherence to the programme. The nature and content of the CBPA programme were what the women desired. However, the schedule of the programme seems to be the main concern of the women, although many of them were housewives, daily household routines occupied most of their time. Besides, most women agreed that the content of the programme addressed their interests and needs and made them decide to join without hesitation.

#### Adherence to the programme

There were four components in the programme, all components were suggested by the women and designed according to women’s interest. This enhanced the adherence of women to the programme, as the whole programme met the women’s interest as a result and most participants completed the whole CBPA programme, as described below:

I joined the whole programme, I loved all the components, those were what I wanted, the schedule was good and the arrangement was nice which I could join at my free time… I appreciated that there were multi-components… it included individual and family components, which I have never seen… (N09)

#### Barriers to join the programme

Few women were unable to complete the whole programme as they were the main care provider in the family, the schedule of each component of the programme might not be able to fit all of them, this seems to be the major factor influencing the feasibility of the programme.

I was unable to join the ‘Making enzyme activity’ because the date was close to Lunar New Year, and I had to do a lot of household jobs, such as cleansing house, making food… I must finish all the jobs otherwise my husband may get angry… (N06)

Another factor which made it difficult to participate in the programme was the women’s physical condition, such as unpredictable physical discomfort. Nevertheless, women found the content of the programme was good enough to address their needs.

I missed the ‘Brain Gym activity’ as I have to attend a medical follow-up; I hope it could be held again so that I can join it. (N03)

### Theme 4: Empowering the women through participating in CBPA

Women who actively engaged in the developmental process of the CBPA programme obtained a sense of achievement and achieved high satisfaction. Immersion in the process enhanced women’s courage as they were able to break through their limitations and challenges and their ability was being recognized. As a result, women were empowered through active participation in the development process of the CBPA programme, as told by the women:

I had low self-confidence before joining this programme. I had several new attempts in this participation… I assisted the tutor to teach other women to make cleansing enzyme, it was my first time to be a tutor’s assistant, I had a strong sense of pride… (N08)

According to the core principles of CBPA, community services providers, researchers and the stakeholders collaborate throughout the process with shared decision-making authority. Autonomy and independence over choices were given to the women during the process, so women derived a sense of power as they felt they were part of the team and trust seems to be a facilitator to empowerment. Through participating in CBPA, it allowed abused women’s voices to be heard directly instead of researchers to translate their voices.

I was so excited and happy that my opinion was being valued and accepted. One of the activities was my idea, this gave me a feeling of identity. (N01)

I feel like my voice is important… (N04)

In addition, women described how this approach made them felt differently when comparing with any other programme they had ever joined. Women had more enjoyment because it provided more autonomy and fewer constraints during the process. Women gained a sense of self-control, felt they were being respected and their self-esteem was raised. They felt free to contribute ideas without any hesitation.

I loved this style (CBPA) and concept of this approach as I can have more control over it. I have freedom to share my thoughts, it was more flexible when comparing with my previous experience,… (N09)

The programme belongs to us, we designed and made it for ourself, there are fewer regulations, I felt so lucky for becoming part of the team, I enjoyed this experience… (N10)

## Discussion

To the best of our knowledge, this is the first study to explore abused Chinese women’s perceptions of CBPA in addressing their needs and their experiences throughout a CBPA process. This qualitative study allows researchers to discover the appropriateness of adopting CBPA in intervention development. Exploring the perceptions from user’s perspectives is essential for understanding the effectiveness of an intervention, in congruences with the principle of CBPA, everything begins from the stakeholder’s perspectives, which is a bottom-up approach. Findings in this study are in line with current literature, showing CBPA is able to address multi-faceted needs of community stakeholders including abused women (Krieger et al., 2002; Poleshuck et al., 2018; Sims-Gould et al., 2017; Yoshihama et al., 2012; Zhou et al., 2016). CBPA allows researchers and service providers to engage more deeply with participants, as their needs could be addressed more effectively and their concerns could be responded more appropriately. The findings in this study provide empirical evidence that the CBPA programme provides a series of beneficial effects on the women, their psychological well-being was improved, their mindset became more positive and psychological stress was relieved. Women had less conflict with their husband and their social networks were enhanced. We learnt from literature that a social network is essential to abused women as the majority of them would seek informal sources of help, having a higher intention to disclose themselves to peers (Djikanović et al., 2012). The findings are compatible with Western literature that a CBPA programme improves physical health and psychological well-being across diverse populations in various ways (Davis et al., 2017; Kobeissi et al., 2011; Ma et al., 2012; Nguyen et al., 2011; Tapp et al., 2013). However, due to the nature of this CBPA programme, which is tailor made and culturally sensitive to the specific population, it is not feasible to compare the specific benefits due to the heterogenous nature of the content of the intervention.

A qualitative approach helps to derive a comprehensive and in-depth understanding of benefits brought to the women. Through descriptions and explanations from the participants, we learn about how each component benefits them and how this provides a mutual effect by interacting with another to generate an additional effect on family harmony. Findings in this study help to advance our understanding of the effect of active engagement in CBPA on the abused Chinese women. There is a dearth of research investigating the effect of CBPA itself, with current studies considering health outcomes as an indicator for the success of the CBPA programme. Research on the impact of the approach on the stakeholders was limited. According to the principles of CBPA, equitable partnership, power sharing and empowerment are the key elements in this approach (Israel et al., 1998). The effect of the CBPA itself should not be ignored and it is worth understanding whether the community stakeholders benefit through the active engagement. Findings of this study revealed that the women were empowered, they felt respected in the process, which is consistent with literature that indicates that a health disparities group of people were empowered by CBPA (Lin et al., 2019; Pham, 2016; Stack & McDonald, 2018; Tapp et al., 2014).

Empowerment of the women was created during their active engagement in the process; this benefit echoed the concept of current interventions for abused women (Rivas et al., 2019). The participants took up special roles and they were fully engaged throughout the entire process, were invited to express their concerns, raised their thoughts and provided input throughout the intervention development process, their voices being acknowledged, their opinions being accepted and their participation helping to shape the content of the intervention. The women worked with researchers and service providers, they became part of the research team, all bringing them benefits. In light of the power-sharing, co-learning and equitable partnership aspects of CBPA (Israel et al., 1998), abused women were engaged and realized their value in the group, which promoted a sense of membership and belonging to the women and apparently that abused Chinese women were empowered through this partnership. Furthermore, the participants had a high level of satisfaction regarding the CBPA programme in terms of the content and approach, as their opinions were taken into consideration. Truly listening to the community stakeholders, getting feedback and incorporating their opinions could enhance the sustainability and effectiveness of the intervention which demonstrated a meaningful participation in the process of the CBPA up to evaluation (Charania & Tsuji, 2012; Tapp et al., 2014).

The majority of the current interventions tend to be of a top-down nature which may run contrary to the concept of empowerment—it would be difficult to address the specific needs of the women because the ideas did not come from those actually using the services. It is not surprisingly that the women have positive feedback on the CBPA programme, it was because the programme was designed based on their preferences, needs and inputs. The abused Chinese women who are the stakeholders actively participated in the intervention development from preparation to implementation. Sullivan and Bybee (1999) suggested that the direction of an intervention should be guided by the participant and Constantino et al. (2005) mentioned that research incorporating women’s input is essential so that it is capable to address their needs, the most effective interventions are those women themselves evaluate or prefer (Constantino et al., 2005).

A bottom-up approach may be able to fill this gap by providing actual empowerment spirit for abused women and result in fulfilling their specific needs through listening to them, offering the women more control in the planning stage and encouraging their participation in the entire process. Giving the abused women opportunity, authority, autonomy and motivation as well as making them responsible and accountable for the outcome of their actions through their contributions could definitely enable them to overcome their sense of powerlessness by recognizing what resources and opportunities they can eventually utilize.

## Implications for practice

Findings of this study inform researchers of the benefits of adopting CBPA in intervention development, especially for future design of interventions. This study serves as an important step in practice to demonstrate the CBPA approach is highly accepted by community-dwelling abused Chinese women. Moreover, the CBPA programme provide various benefits in terms of physical and psychological health to the women. Further study of CBPA intervention for abused Chinese women can be considered to be scaled up as part of regular public health intervention, so more women could benefit. Multi-disciplinary collaboration is recommended owing to the multi-faceted needs of the women and diverse components, as suggested in the CBPA programme. Adopting such an approach in intervention development can be an option for health promotion and health education programmes, diseases prevention activities, especially chronic diseases, which may enhance adherence and maximize sustainability and success of the intervention. Participants being empowered become an essential element for the success of health promoting intervention, especially for socially disadvantaged groups. Researchers have great responsibility in the violence against women issue, rising this approach to be able to address the complex health related issue in a more effective way.

## Limitations

This is the first study using a qualitatively approach to explore abused Chinese women’s perceptions of CBPA as well as their experiences. Nevertheless, several limitations are noted from this study. First, only abused Chinese women from a community setting were recruited in this study, findings which may not be applicable to those in other settings. Second, this approach seems to be acceptable and appropriate to be used in abused Chinese women, however, culture appropriateness in other ethnic groups should be further investigated. Furthermore, the qualitative study design was appropriate in eliciting rich information about the women’s perceptions and experiences, however, women’s satisfaction and effectiveness of their chosen interventions could not be objectively measured.

## Conclusion

This study provided new insights for researchers that a community-based participatory approach programme was perceived as empowering and beneficial to the abused Chinese women in various aspects and able to address their multi-faceted needs. A community-based participatory approach may be a promising approach considered as a single approach or incorporated into current practice as a combined approach to strengthen the design of future intervention for addressing the multi-faceted needs of community-dwelling abused Chinese women as well as enhancing the effectiveness of intervention. More objective findings to support the benefits of adopting CBPA for the abused women warrants further research.
